# Prebiotics, probiotics, and synbiotics and maternal mental health during pregnancy and postpartum period: a systematic review

**DOI:** 10.3389/fnut.2026.1776398

**Published:** 2026-02-17

**Authors:** Sandra Martín-Peláez, Luis Miguel Martín-delosReyes, Naomi Cano-Ibáñez

**Affiliations:** 1 Department of Preventive Medicine and Public Health, Faculty of Medicine, University of Granada, Granada, Spain; 2 CIBER Epidemiología y Salud Pública (CIBERESP), Instituto de Salud Carlos III (ISCIII), Madrid, Spain; 3 Instituto de Investigación Biosanitaria de Granada (ibs.GRANADA), Granada, Spain

**Keywords:** anxiety disorder, depressive disorder, microbiota, postpartum, pregnancy

## Abstract

**Background:**

Depression and anxiety are common during pregnancy and postpartum, affecting both mothers’ and offspring health. Emerging research suggests gut microbiota may influence these conditions, providing a potential non-pharmacological approach for primary prevention, particularly in women without a prior mental health diagnosis.

**Aim:**

To assess the effect of prebiotics, probiotics, and synbiotics as dietary interventions targeting gut microbiota for preventing mental health disorders during pregnancy and postpartum in women without diagnosed mental health disorders.

**Methods:**

The protocol was prospectively registered in PROSPERO (CRD42024576678). A comprehensive search of MEDLINE, EMBASE, CINAHL, The Cochrane Library (CENTRAL), Scopus, and Web of Science from inception to March 2025, without language restriction. Randomized controlled trials (RCTs) involving pregnant or early postpartum women without a diagnosed mental health disorder, evaluating prebiotics, probiotics, or synbiotics and reporting maternal mental health outcomes, were included. Risk of bias was assessed using the Cochrane Risk of Bias 2 tool.

**Results:**

Of the 1,401 records identified, four RCTs (*n =* 1,342 women) met the inclusion criteria. All RCTs evaluated probiotics interventions, none assessed prebiotics or synbiotics. Of four, two RCTs using *Lactobacillus rhamnosus* HN001, *Limosilactobacillus reuteri* PBS072 and *Bifidobacterium breve* reported small but significant reductions in depressive symptoms [Edinburgh Postnatal Depression Scale, MD = −1.2; 95% CI (−2.3, −0.1)] and anxiety symptoms [State–Trait Anxiety Inventory-6: MD = −1.0; 95% CI (−1.9, −0.2); *p* < 0.05)] during pregnancy. One RCT showed significantly lower depressive scores in the intervention group at day 45 (mean 9.0 ± 4.8 vs. 12.1 ± 5.9; *p* < 0.001) and day 90 (7.0 ± 3.3 vs. 10.8 ± 6.2; *p* < 0.001) of postpartum compared to placebo. No pooled analyses were conducted due to heterogeneity. Risk of bias was moderate in three RCTs and high in one, primarily due to selective outcome reporting.

**Conclusion:**

Evidence on the effect of probiotics for preventing maternal mental health disorders during pregnancy and postpartum is limited. There were no data on prebiotics or synbiotics. Strain specific probiotic effectiveness studies, as well as studies on prebiotics and synbiotics are required in the future.

**Systematic review registration:**

PROSPERO, identifier (CRD42024576678).

## 1 Introduction

Maternal mental health disorders affect about one in five women during pregnancy and the first postpartum year, with depression and anxiety being most common conditions (1). These disorders are linked to adverse outcomes for both maternal well-being and infant development (2). Although pharmacotherapy is a mainstay treatment, concerns about maternal and fetal safety have increased interest in alternative approaches (3). Non-pharmacological interventions, such as cognitive-behavioural therapy and exercise, have shown potential benefits (4). Recent studies suggest a relationship between maternal mental health disorders and the gut microbiota through the gut–brain axis (5). The gut microbiota influences the central nervous system via autonomic, hormonal and immune pathways and has been associated with psychological disorders (6–8). Pregnancy induces physiological changes in gut microbiota composition (9, 10), while environmental disturbances may negatively affect maternal biology and mental health (11, 12). Maternal microbiota perturbations may influence offspring development through microbiota-derived fetal programming and early-life microbial transmission from mother to infant (13, 14).

Importantly, gut microbiota composition is modifiable through lifestyle factors, particularly diet. Dietary patterns, specific nutrients, and microbiota-targeted interventions such as probiotics, prebiotics and synbiotics have shown mental health benefits (15–18). Despite growing interest in microbiota-targeted interventions, evidence on their role in primary prevention of maternal mental health problems remains limited (19–21). In this context, prevention refers to interventions applied in women without a prior clinical diagnosis, aimed at reducing symptom severity or the risk of progression to clinically relevant disorders, rather than preventing formally diagnosed conditions. Previous systematic reviews have reported heterogeneous findings and methodological limitations, and none incorporated prebiotic or synbiotic interventions (19, 20). Moreover, their methodological quality assessed using AMSTAR-2 was low (Supplementary material 1). These limitations justify the need for an updated systematic review to evaluate current evidence and identify research gaps.

This systematic review aims to evaluate the available evidence and identify research gaps regarding the effect of prebiotics, probiotics, and synbiotics as dietary interventions targeting gut microbiota to prevent mental health disorders during pregnancy and postpartum in women without diagnosed mental health disorders.

## 2 Methods

This systematic review was carried out in accordance to Preferred Reporting Items for Systematic Reviews and Meta-Analyses (PRISMA) statement (22). A protocol for this review was registered in the International prospective register of systematic reviews (PROSPERO ID: CRD42024576678).

### 2.1 Search strategy and selection criteria

A comprehensive bibliographic search was conducted (Supplementary material 2) across the electronic databases, MEDLINE, EMBASE, CINAHL, The Cochrane Library (CENTRAL), Scopus, Proquest Dissertations and Theses, and Web of Science Core Collection, from inception to March 2025. The search strategy, developed by a health sciences librarian (CHC), focused exclusively on randomized controlled trials (RCTs), excluding animal studies. No restrictions on language or publication date were applied.

Only randomized controlled trials (RCTs) evaluating prebiotic, probiotic, and/or synbiotic supplementation (PPS) conducted in pregnant women without a diagnosis of mental health disorder were included. The absence of a mental health disorder was defined according to the inclusion criteria of each individual trial and was generally based on self-report and/or the absence of a prior clinical diagnosis, rather than on structured psychiatric interviews. The presence of subthreshold depressive or anxiety symptoms was not an exclusion criterion in most studies, which is consistent with a primary prevention framework but limits conclusions regarding clinically diagnosed disorders.

The intervention of interest was PPS, alone or in combination compared to treatment as usual, standard advice or a placebo control group. The primary outcomes reviewed were measures of maternal mental health (well-being, anxiety, depression, stress, mood, nervous, obsession, etc. For more terms used, see Supplementary material 2) during pregnancy and the postpartum period. As secondary outcomes, health problems and changes in the gut microbiota of the mother or offspring were reviewed. Review articles, editorials, letters, case series, case reports, quasi-experimental studies and observational studies were excluded from the review.

After duplicates were removed, the selection process was conducted in two phases: first, titles and abstracts were screened for relevance, followed by a review of the full text to ensure that the inclusion criteria were met. Two independent reviewers (LMMR and SMP) carried out the screening process with the help of the Rayyan application (23). Disagreements between reviewers were resolved by discussion or, when necessary, by consulting a third reviewer to reach consensus (NCI). Reasons for exclusion were further documented (Supplementary material 3).

### 2.2 Data extraction

Data extraction was performed by two independent reviewers (LMMR and SMP) using a standardized form. The following information was collected for each included study: study characteristics (author, year of publication, setting, sample size), participant characteristics (age, gestational stage), intervention details (type, dose, duration of probiotics, prebiotics or synbiotics intervention in experimental and control group), primary outcome (type of mental disorder and measurement tool), secondary outcomes (type of health disorder/changes in maternal or newborn gut microbiota and measurement tool). Reported side effects or adverse reactions were also recorded. All reported mean differences (MDs) correspond to individual study estimates, as reported in the original publications. Results were synthesized using a structured narrative approach, with findings presented at the individual study level.

Discrepancies between reviewers (LMMR and SMP) were resolved by discussion. When consensus was not reached, a third reviewer (NCI) was consulted to ensure accurate and comprehensive data extraction.

The quality of the included studies was assessed using the Risk of Bias 2 (ROB2) tool (24). This tool assesses the risk of bias in RCTs in five domains: Randomization process, Deviations from intended interventions, Missing outcome data, Outcome measurement, and Selection of reported outcome. Each domain was classified as “low risk,” “some concern,” or “high risk” based on the criteria described in the ROB2 tool. The overall risk of bias for each study was determined by considering the ratings across all domains. This quality assessment was performed by two independent reviewers (LMMR and SMP), and disagreements between reviewers were resolved by discussion or, when necessary, by consulting a third reviewer to reach consensus (NCI).

### 2.3 Evidence synthesis

We tabulated the findings and constructed graphs as appropriate. Due to small number of studies, statistical synthesis was not performed.

## 3 Results

**Figure 1:**
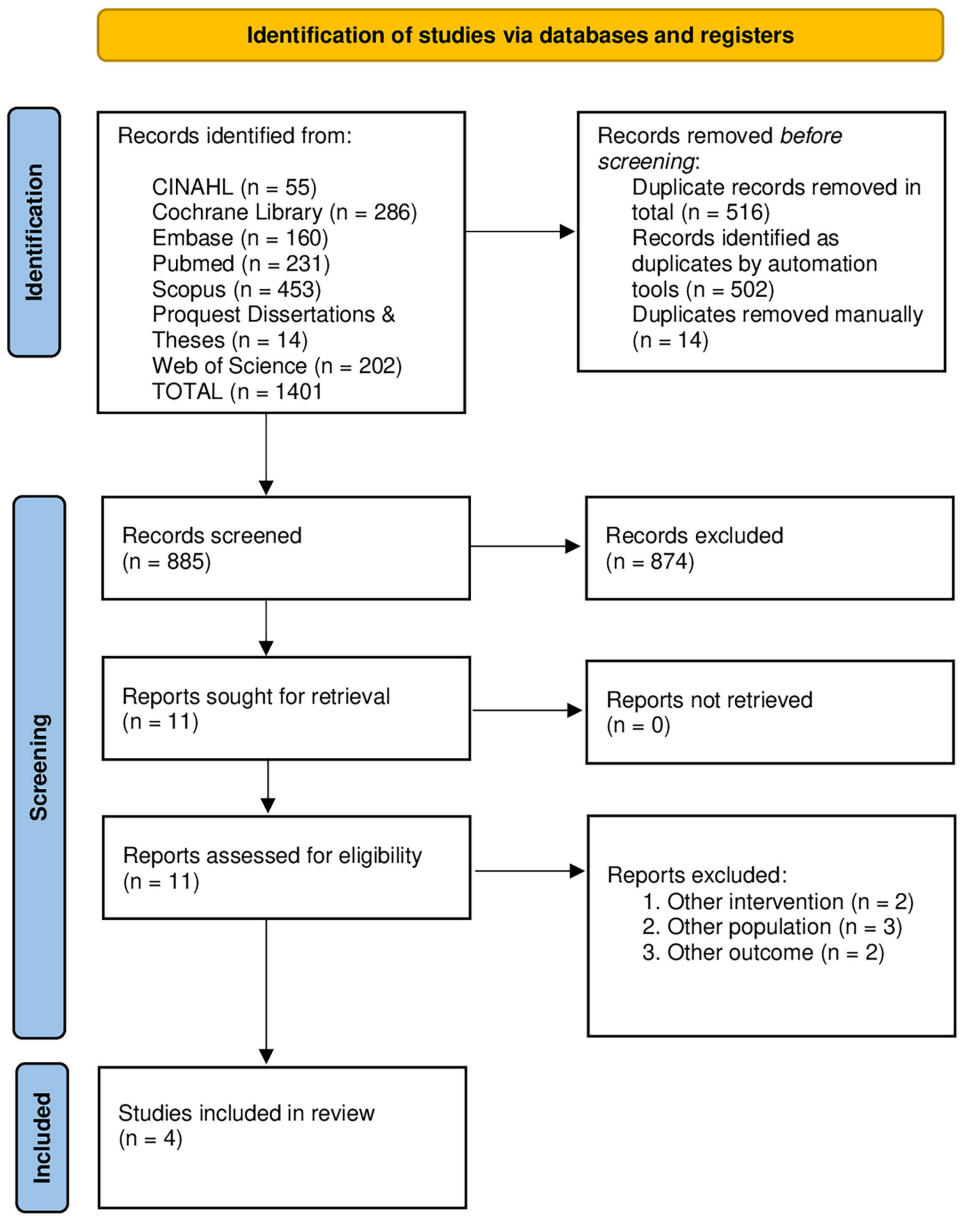
PRISMA 2020 flow diagram for the systematic reviews including searches of databases, registers, and other sources.

A total of 1,401 records were identified through database searches. After screening titles and abstracts, 11 records were assessed for eligibility. Of these, 6 were excluded based on predefined inclusion criteria, remaining 5 full-text articles for further evaluation. Ultimately, after removal of one study because it included women diagnosed with a mental health disorder, 4 studies met all inclusion criteria and were included in this systematic review (Figure 1).

## 4 Characteristics and quality of included studies

The included studies were carried out in New Zealand (25), Finland (26), Iran (27), and Italy (28). The publication dates ranged from 2016 to 2023. The total number of participants varied from 60 (27) to 439 (26), with a cumulative total of 1,342 women included in this review. The gestational age at the start of the trials did not exceed 28 weeks, and the participants’ ages ranged from 16 (25) to 50 years (28) (Table 1). The four RCTs included probiotics in the interventions. The probiotic strains used were *Lactobacillus rhamnosus* HN001 (25, 26), *Bifidobacterium animalis ssp. lactis* 420 (26), *Limosilactobacillus reuteri* PBS072 (28), *Bifidobacterium breve* BB077 (28), *Lactobacillus acidophilus* La-5 and *Bifidobacterium lactic* Bb-12 (27). The probiotic daily dose varied from 4 × 10^9^ CFU (28) to 4.8 × 10^10^ CFU (27), administered in capsules with the exception of one study, which used yogurt as probiotic vehicle (27). Intervention duration ranged from 4 weeks (27) to 48 weeks (25), and occurred during pregnancy only (27), both pregnancy and postpartum (25, 26) or only postpartum (28). From the four studies, three investigated mental health as primary outcome (25, 26, 28). The mean intervention follow-up rated from 12 to 24 months (Table 2).

**Table 1:** Characteristics of the studies.

Author, year	Country	RCT design	Population		
			Sample Size (intervention/Control)	Inclusion criteria	Exclusion criteria
Hulkkonen et al. (26)	Finland	Double-blind, 2×2 factorial parallel groups	*N =* 439 (split into 4 groups)	1) Age: Not specified 2) Type of pregnancy: Singleton 3) GW: ≤ 18 weeks of gestation 4) BMI: ≥ 25 Kg/m2 5)Absence of chronic diseases (except for asthma and allergies)	Multifetal pregnancy, the presence of inflammatory, metabolic or gastrointestinal diseases diagnosis or history of coagulopathy, and use of anticoagulants. The women who became pregnant again before 12 months postpartum were excluded from the postpartum analyses
Mirghafourvand et al. (27)	Iran	Triple-blind, two parallel groups	*N =* 60 (30/30)	1) Age: >18 years 2) GW: between 24–28 weeks of gestation 4) Women suffered from constipation 5) Being literate and willing to participate in the study	Receiving any treatment in less than a week before the study, having mental retardation or metabolic disease (hypothyroidism), Hirschsprung disease, spinal anomalies, anorectal pathology, inflammatory of bowel disease, previous gastrointestinal surgery and use of fermented dairy products containing probiotics 2 weeks prior to the study
Slykerman et al. (25)	New Zealand	Double-blind, two center	*N =* 423 (212/211)	1) Age: >16 years 2) GW: between 24–28 weeks of gestation 4) English-speaking 5) Planning to breastfeed 6) They or the unborn child’s biological father had a history of asthma, hay fever or eczema requiring medication	Women were excluded from the study if aged < 16 years, planning to move outside the study centers during study duration, planning on taking probiotics, or if they had serious medical or health problems related to the pregnancy
Vicariotto et al. (28)	Italy	Double-blind, multicenter, two parallel groups	*N =* 190 (95/95)	1) Age 18–50 years old 2) Women in the first trimester postpartum 3) Good general health condition 4) Willingness to: breastfeed, use probiotics and multivitamin food supplements consigned at the last visit before delivery, fill up questionnaires, use only the products to be tested during the entire study period, not to use similar products that could interfere with the product to be tested, not to vary the normal daily routine (i.e., lifestyle, physical activity, etc.) 5) Subjects aware of this study’s procedures and having signed an informed consent form	1) Subjects who do not meet the inclusion criteria 2) Subjects considered as not adequate to participate in this study by the investigator 3. Subjects with known or suspected sensitization to one or more test formulation ingredients 4) Adult protected by law (under control or hospitalized in public or private institutions for reasons other than research or incarcerated) 5) Subjects not able to communicate or cooperate with the investigator due to problems related to language, mental retardation, or impaired brain function 6) Subjects suffering from other psychiatric disorders such as schizophrenia, other psychotic disorders, bipolar disorder, or substance use disorder 7) Subjects with serious physical illnesses or mental disorders 8) Subjects with significant risk of infanticide according to the investigator’s assessment 9) Subjects taking herbal remedies or psychotropic drugs intended for depression and taken within the last 2 weeks prior to baseline or during this study 10) Subjects receiving counselling or psychological therapies at baseline or during this study

**Table 2:** Summary of main results.

Author, year	Intervention				Control**	Mental health outcomes			
	Type	Bacterial strains	Dose	Duration	Type	Type	Test	Period	Results
Hulkkonen et al. (26)	Fish oil and Probiotics*	*Lactobacillus rhamnosus* HN001 and *Bifidobacterium animalis ssp. lactis* 420	1 capsule/day containing 10^10 CFU per strain	Enrolment (≤18 GW) to 6 months postpartum	Placebo for the probiotics: capsule containing microcrystalline cellulose Placebo for fish oil: capsule containing medium chain fatty acids	Depressive and anxiety symptoms	EPDS (Ediburgh Posnatal Depression Scale) SCL-90 (Symptom Checklist-90)	Early pregnancy and late pregnancy and at three, six and 12 months postpartum	No differences among intervention groups and placebo
Mirghafourvand et al. (27)	Probiotics	*Bifidobacterium lactic* (Bb-12) and *Lactobacillus acidophilus* (La-5)	300 grams of yogurt containing 4.8 × 10^10 CFU (combined strains), 100 g x three times a day	Four weeks	Conventional yogurt, identical appearance	Quality of life	SF-36 (Quality of life questionnaire)	At baseline and at the end of week 6	No differences among intervention group and placebo
Slykerman et al. (25)	Probiotic	*Lactobacillus rhamnosus* HN001	1 capsule/day containing 6 × 10^9 CFU	Enrolment until birth and, from birth up till 6 months post-birth whilst breastfeeding	Capsules containing corn-derived maltodextrin, identical appearance	Depressive and anxiety symptoms	EPDS (Ediburgh Posnatal Depression Scale) STAI-6 (State Trait Anxiety Inventory-6)	Mothers (baseline and after 6 months or 12 months after delivery) completed the surveys based on how they remember they felt when their child was 1–2 months old	Depression: The intervention group reported significantly lower scores (7.7 ± 5.4) than placebo (9.0 ± 6.0). Mean difference −1.2 (−2.3, −0.1), *p* = 0.037 Anxiety: The intervention group also reported lower scores (12.0 ± 4.0 vs. 13.0 ± 4.3). Mean difference: −1.0 (−1.9, −0.2), *p* = 0.014
Vicariotto et al. (28)	Probiotics and plus multivitamin food supplement	*Limosilactobacillus reuteri* PBS072 and *Bifidobacterium breve* BB077	1 capsule/day containing 4 × 10^9 CFU/day (2 × 10^9 CFU for each strain)	90 days	The same capsules without the probiotic strains (identical appearance)	Postpartum depression symptoms	EPDS (Ediburgh Posnatal Depression Scale)	45 days after the beginning of the treatment and final visit (i.e., 90 days after the beginning of the treatment)	At day 45, the intervention group showed a significantly lower mean score (9.0 ± 4.8) compared to the control group (12.1 ± 5.9) (*p* < 0.001). On day 90, the intervention group showed a significantly lower mean score (7.0 ± 3.3) compared to the control group (10.8 ± 6.2) (*p* < 0.001).

*In this study, the two interventions were combined in four groups with the control, resulting in the following organization: (1) Fish oil + placebo, (2) probiotics + placebo, (3) fish oil + probiotics and (4) placebo + placebo. ** In all studies, the duration and dose of placebos was identical to the intervention.

**Figure 2:**
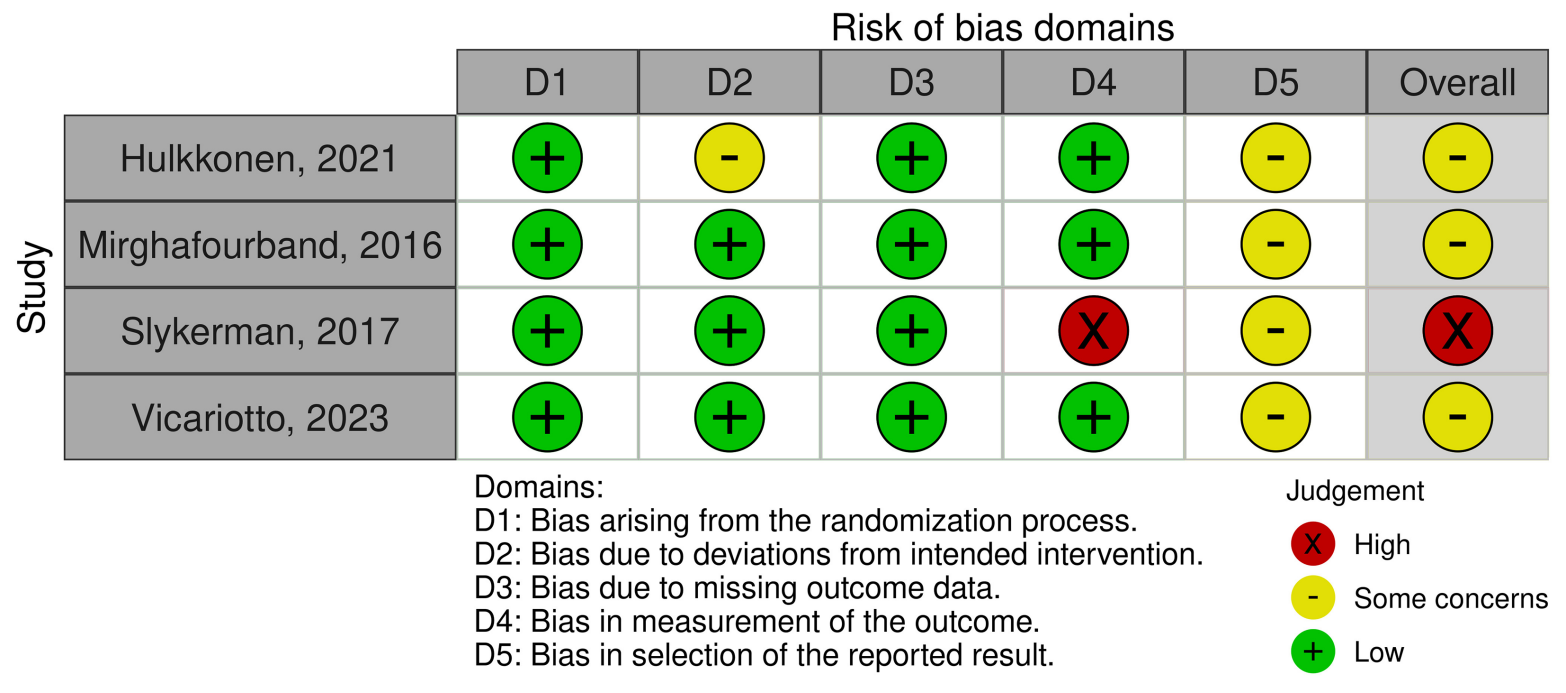
Risk of bias assessment of the included studies across individual domains and overall judgment.

**Figure 3:**
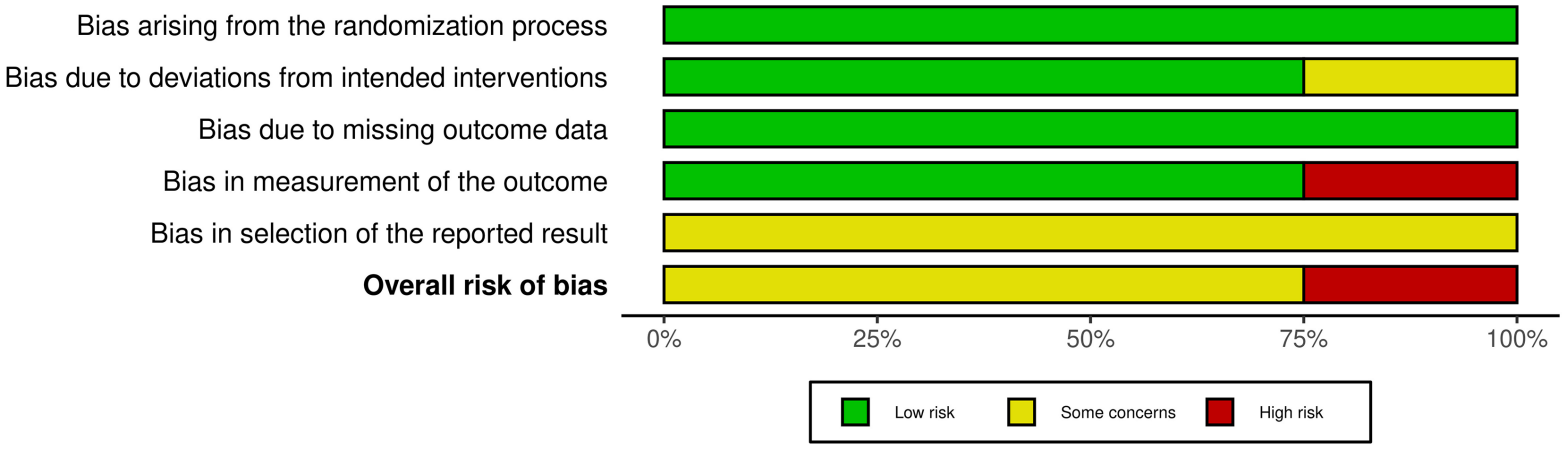
Summary of overall risk of bias across domains for the included studies.

Of the included studies, one was classified as a high risk (Figure 2), the remaining 3 studies were classified as moderate. Among the ROB2 domains (Figure 3), the most frequent critical weakness identified was the risk of bias in the selection of the reported outcome.

### 4.1 Primary outcomes

Depressive (*n =* 3) and anxiety (*n =* 2) symptoms were the most frequently assessed outcomes, measured using tools such as the Edinburgh Postnatal Depression Scale (EPDS) (25, 26, 28), the State Trait Anxiety Inventory-6 (STAI-6) (25) and the Symptom Checklist-90 (SCL-90) (26). In addition, one study measured quality of life using the quality-of-life questionnaire (SF-36) (27), which assesses aspects such as emotional problems, emotional well-being and related domains (Table 2).

Regarding depressive or anxiety symptoms during pregnancy, one study (26) found not statistically significant differences between intervention *Lactobacillus rhamnosus* HN001 plus *Bifidobacterium animalis ssp. lactis* 420 and control groups, although minor variations were observed at 12 months of follow-up in EPDS test scores between the intervention groups containing fish oil and placebo versus probiotics and placebo (MD = −1.12, *p* = 0.039). In contrast, another study (25) reported significant reductions in depression mean differences (MD) scores in the intervention group (*Lactobacillus rhamnosus* HN001) compared to the control [MD = −1.2 95% CI (−2.3, −0.1)] and anxiety mean scores [MD = −1.0 95% CI (−1.9, −0.2)]. Regarding postpartum depression, one study (28) reported that at day 45, the intervention group (*Limosilactobacillus reuteri* PBS072 plus *Bifidobacterium breve* BB077) showed a significantly lower mean score (9.0 ± 4.8) compared to the control group (12.1 ± 5.9) (*p* < 0.001) and at day 90 (7.0 ± 3.3 vs. 10.8 ± 6.2, *p* < 0.001) (Table 2). Another study (27) showed not significant differences between intervention (*Lactobacillus acidophilus* La-5 plus *Bifidobacterium animalis subsp. lactis* Bb-12) and control groups regarding quality of life.

### 4.2 Secondary outcomes

From the 4 studies included in the review, only one (28) reported information about the secondary outcomes. Using the Breastfeeding Self-Efficacy Scale—Short Form (BSES-SF), they found that 45 and 90 days after the beginning of the intervention, breastfeeding quality and the baby’s crying/fussing significantly improved in the probiotic group. At 45 days postpartum, 81% of mothers in the intervention group reported improvement in crying/fussing events compared with 42% in the control group, while at 90 days postpartum the corresponding figures were 78 and 43%, respectively. In addition, the mean daily number of crying/fussing events was significantly lower in the probiotic group compared with the control group at both time points (*p* < 0.001).

## 5 Discussion

The evidence obtained in this study on the effect of prebiotics, probiotics, and synbiotics for preventing maternal mental health disorders is limited and heterogeneous. Only four RCTs met inclusion criteria, all focused on probiotics, highlighting a major research gap regarding prebiotics and synbiotics (19, 20). Importantly, by explicitly including prebiotics and synbiotics in the research question and search strategy, this review provides an updated assessment of the current evidence base and clearly demonstrates the absence of eligible RCTs for these interventions in perinatal mental health. While two trials reported improvements in depressive and anxiety symptoms (25, 28) others found no significant effects on mental health outcomes or quality of life (26, 27), in line with previous systematic reviews showing mixed results (19–21).

Importantly, the available evidence pertains to symptom-based outcomes rather than clinically diagnosed mental health disorders. Most studies allowed the presence of subthreshold symptoms and relied on self-reported measures rather than structured diagnostic interviews. Therefore, findings should be interpreted within a primary prevention framework focused on symptom reduction, rather than prevention of diagnosed mental health disorders.

Although secondary outcomes were reported in only one included trial, breastfeeding-related outcomes and infant crying/fussing are indirectly relevant to maternal mental health in the postpartum period. Postpartum depressive symptomatology has been consistently associated with less favorable infant-feeding outcomes, including breastfeeding difficulties and lower breastfeeding self-efficacy (29, 30). Moreover, evidence from systematic reviews and cohort studies indicates that excessive or inconsolable infant crying is associated with maternal depressive and anxiety symptoms, suggesting bidirectional and stress-mediated pathways (31–33). Nevertheless, because these secondary outcomes were reported in only one included trial, they should be interpreted cautiously and cannot be generalized.

Methodological heterogeneity likely explains these discrepancies, including wide variation in participant age (25, 28), intervention duration (27), probiotic strains and doses (34), and outcome measures. Some strains, such as *Lactobacillus rhamnosus* HN001 showed promising effects (25), but findings remain inconclusive due to limitations such as retrospective data collection and high dropout rates (26, 35). Other strains demonstrated benefits in specific populations or contexts (28, 36–40), suggesting context- and strain-specific effects.

Additional limitations include lack of standardized dosing (41), inadequate control of antibiotic use by participants in RCTs (25, 26, 28), reliance on self-reported adherence (42, 43), high microbiota variability (44, 45), heterogeneity in psychological instruments with varying validity (25, 26, 46–52) and absence of structured clinical interviews (53). Together, these issues underscore the need for well-designed, standardized RCTs to clarify the role of probiotics in maternal mental health.

### 5.1 Strengths and limitations

This systematic review followed PRISMA guidelines and was prospectively registered in PROSPERO, ensuring transparency and reproducibility. The search strategy was comprehensive, developed with a health sciences librarian, and applied across multiple databases without language or date restrictions. Study selection, data extraction, and risk of bias assessment (ROB2) were performed independently by two reviewers, with disagreements resolved by a third reviewer.

Limitations mainly reflect the scarcity and heterogeneity of the available evidence. Only four RCTs were identified, all evaluating probiotics, with no eligible trials assessing prebiotics or synbiotics. Differences in probiotic strains, doses, intervention duration, and outcome measures limited comparability across studies and precluded meta-analysis. Finally, the limited geographic representation may reduce generalizability to other populations and settings.

## 6 Conclusion

Current evidence on the effect of probiotics for preventing mental health disorders during pregnancy and postpartum is limited and heterogeneous, with no RCTs evaluating prebiotics or synbiotics. Although some studies report symptoms reductions, heterogeneity in strains, doses, intervention duration, and measurement tools limit conclusions. Well-designed and adequately powered RCTs are needed where maternal depressive and anxiety symptoms are primary outcomes, using standardized and strain-specific interventions, appropriate doses and durations, and harmonized assessment tools. Future trials should also explicitly evaluate prebiotics and synbiotics, as these remain major evidence gaps in perinatal mental health research.
