# Supplementary material for: Prebiotics, probiotics, and synbiotics and maternal mental health during pregnancy and postpartum period: a systematic review
Source: Front Nutr. 2026 Feb 17;13:1776398. doi: 10.3389/fnut.2026.1776398 (PMC12955548; doi:10.3389/fnut.2026.1776398)
Supplement: Supplementary file 1 [file Table_1.docx]

Supplementary Material

**Supplementary Material 1. AMSTAR-2.**

|  |  | **Quality Assessment** | | | | | | | | | | | | | | | | |
| --- | --- | --- | --- | --- | --- | --- | --- | --- | --- | --- | --- | --- | --- | --- | --- | --- | --- | --- |
|  |  | 1) Aim and inclusion criteria include integrity issue, 2) Prospective registration, 3) Selection of the studies for inclusion, 4) Literature search strategy, 5) Duplicated study selection, 6) Duplicated data extraction, 7) List of excluded studies and exclusions, 8) Integrity issue description, 9) Technique for assessing risk of bias, 10) Funding of the included studies, 11) Statistical combination of results (if meta-analysis), 12) Risk of bias incorporated in meta-analysis, 13) Risk of bias considered in interpreting/discussing results, 14) Discussion on heterogeneity, 15) Assessment of publication bias, 16) Conflict of interest. | | | | | | | | | | | | | | | | |
| **Author** | **Year** | **1** | **2** | **3** | **4** | **5** | **6** | **7** | **8** | **9** | **10** | **11** | **12** | **13** | **14** | **15** | **16** | **Total** |
| **Desai V** | **2021** | Yes | Yes | Yes | Yes | Yes | Yes | PY | Yes | Yes | Yes | Yes | Yes | Yes | Yes | No | Yes | Low |
| **Trifkovič K** | **2022** | Yes | No | Yes | PY | No | Yes | PY | Yes | Yes | No | Yes | No | No | No | No | Yes | CL |
| **Halemani K** | **2023** | No | Yes | No | Yes | No | Yes | PY | Yes | Yes | No | Yes | No | Yes | No | No | Yes | CL |

PY: Probably yes. CL: Critically low

**Supplementary Material 2. Systematic review search strategy.**

| **Database** | **Search Strings** |
| --- | --- |
| **PubMed** | (("Prenatal Care"[MeSH Terms] OR "Maternal Health"[MeSH Terms] OR "Postpartum Period"[MeSH Terms] OR "Pregnancy"[MeSH Terms] OR "Breast Feeding"[MeSH Terms] OR ("pregnan*"[Text Word] OR "prenatal"[Text Word] OR "gestation*"[Text Word] OR "pospartum"[Text Word] OR "puerperium"[Text Word] OR "perinatal"[Text Word] OR "mother*"[Text Word] OR "maternal"[Text Word] OR "breastfeed*"[Text Word])) AND ("Prebiotics"[MeSH Terms] OR "Probiotics"[MeSH Terms] OR "Synbiotics"[MeSH Terms] OR "Lactobacillales"[MeSH Terms] OR "Lactobacillus"[MeSH Terms] OR "Bifidobacterium"[MeSH Terms] OR "Saccharomyces"[MeSH Terms] OR "lactococcus"[MeSH Terms] OR "Bacillus"[MeSH Terms] OR "Lacticaseibacillus"[MeSH Terms] OR "Oligosaccharides"[MeSH Terms] OR "Inulin"[MeSH Terms] OR "Fructooligosaccharide"[Supplementary Concept] OR "Xylooligosaccharide"[Supplementary Concept] OR ("probiotic*"[Text Word] OR "prebiotic*"[Text Word] OR "synbiotic*"[Text Word] OR "psychobiotic*"[Text Word] OR "lactobacil*"[Text Word] OR "bifidobacter*"[Text Word] OR "saccharomyc*"[Text Word] OR "lactococc*"[Text Word] OR "Bacillus"[Text Word] OR "pediococc*"[Text Word] OR "leuconostoc*"[Text Word] OR "lactic-acid-bacteria"[Text Word] OR "Lacticaseibacillus"[Text Word] OR "lactiplantibacillus"[Text Word] OR "levilactobacillus"[Text Word] OR "ligilactobacillus"[Text Word] OR "limosilactobacillus"[Text Word] OR "oligosaccharid*"[Text Word] OR "fructo*"[Text Word] OR "galacto*"[Text Word] OR "oligosaccharide*"[Text Word] OR "xylooligosaccharide*"[Text Word] OR "Inulin"[Text Word] OR "fructans"[Text Word] OR "oligofructose"[Text Word] OR "resistant starch"[Text Word])) AND ("Mental Health"[MeSH Terms] OR ("mental"[Text Word] OR "psychol*"[Text Word] OR "psychiatric*"[Text Word] OR "behavior*"[Text Word] OR "behaviour*"[Text Word] OR "wellness"[Text Word] OR "well-being"[Text Word] OR "wellbeing"[Text Word] OR "anxiety"[Text Word] OR "depression"[Text Word] OR "stress"[Text Word] OR "mood"[Text Word] OR "nervous*"[Text Word] OR "obsesi*"[Text Word])) AND "trial*"[Text Word]) AND (humans[Filter]) |
| **Embase** | (pregnan* OR 'prenatal'/exp OR prenatal OR gestation* OR pospartum OR 'puerperium'/exp OR puerperium OR perinatal OR mother* OR 'maternal'/exp OR maternal OR 'breast feeding education'/exp OR 'breast feeding education') AND (probiotic* OR prebiotic* OR synbiotic* OR psychobiotic* OR lactobacil* OR bifidobacter* OR saccharomyc* OR lactococc* OR 'bacillus'/exp OR bacillus OR pediococc* OR leuconostoc* OR 'lactic-acid-bacteria'/exp OR 'lactic-acid-bacteria' OR 'lacticaseibacillus'/exp OR lacticaseibacillus OR 'lactiplantibacillus'/exp OR lactiplantibacillus OR 'levilactobacillus'/exp OR levilactobacillus OR 'ligilactobacillus'/exp OR ligilactobacillus OR 'limosilactobacillus'/exp OR limosilactobacillus OR oligosaccharid* OR fructo* OR galacto* OR oligosaccharide* OR xylooligosaccharide* OR 'inulin'/exp OR inulin OR 'fructans'/exp OR fructans OR 'oligofructose'/exp OR oligofructose OR 'resistant starch'/exp OR 'resistant starch') AND (mental OR psychol* OR psychiatric* OR behavior* OR behaviour* OR 'wellness'/exp OR wellness OR 'well being'/exp OR 'well being' OR 'wellbeing'/exp OR wellbeing OR 'anxiety'/exp OR anxiety OR 'depression'/exp OR depression OR 'stress'/exp OR stress OR 'mood'/exp OR mood OR nervous* OR obsesi*) AND trial* AND [humans]/lim AND [embase]/lim NOT ([embase]/lim AND [medline]/lim) |
| **CENTRAL** | pregnan* OR prenatal OR gestation* OR pospartum OR puerperium OR perinatal OR mother* OR maternal OR breastfeed*):ti,ab,kw AND (probiotic* OR prebiotic* OR synbiotic* OR psychobiotic* OR lactobacil* OR bifidobacter* OR saccharomyc* OR lactococc* OR bacillus OR pediococc* OR leuconostoc* OR "lactic-acid-bacteria" OR lacticaseibacillus OR lactiplantibacillus OR levilactobacillus OR ligilactobacillus OR limosilactobacillus OR oligosaccharid* OR fructo* OR galacto* OR oligosaccharide* OR xylooligosaccharide* OR Inulin OR fructans OR oligofructose OR "resistant starch"):ti,ab,kw AND (mental OR psychol* OR psychiatric* OR behavior* OR wellness OR well-being OR wellbeing OR anxiety OR depression OR stress OR mood OR nervous* OR obsesi*):ti,ab,kw |
| **CINAHL** | ( pregnan* OR prenatal OR gestation* OR pospartum OR puerperium OR perinatal OR mother* OR maternal OR breastfeed* ) AND ( probiotic* OR prebiotic* OR synbiotic* OR psychobiotic* OR lactobacil* OR bifidobacter* OR saccharomyc* OR lactococc* OR bacillus OR pediococc* OR leuconostoc* OR "lactic-acid-bacteria" OR lacticaseibacillus OR lactiplantibacillus OR levilactobacillus OR ligilactobacillus OR limosilactobacillus OR oligosaccharid* OR fructo* OR galacto* OR oligosaccharide* OR xylooligosaccharide* OR Inulin OR fructans OR oligofructose OR "resistant starch" ) AND ( mental OR psychol* OR psychiatric* OR behavior* OR behaviour* OR wellness OR well-being OR wellbeing OR anxiety OR depression OR stress OR mood OR nervous* OR obsesi* ) AND trial* |
| **SCOPUS** | ( TITLE-ABS-KEY ( pregnan* OR prenatal OR gestation* OR pospartum OR puerperium OR perinatal OR mother* OR maternal OR breastfeed* ) AND TITLE-ABS-KEY ( probiotic* OR prebiotic* OR synbiotic* OR psychobiotic* OR lactobacil* OR bifidobacter* OR saccharomyc* OR lactococc* OR bacillus OR pediococc* OR leuconostoc* OR "lactic-acid-bacteria" OR lacticaseibacillus OR lactiplantibacillus OR levilactobacillus OR ligilactobacillus OR limosilactobacillus OR oligosaccharid* OR fructo* OR galacto* OR oligosaccharide* OR xylooligosaccharide* OR inulin OR fructans OR oligofructose OR "resistant starch" ) AND TITLE-ABS-KEY ( mental OR psychol* OR psychiatric* OR behavior* OR behaviour* OR wellness OR well-being OR wellbeing OR anxiety OR depression OR stress OR mood OR nervous* OR obsesi* ) AND TITLE-ABS-KEY ( trial* ) ) |
| **WOS** | pregnan* OR prenatal OR gestation* OR pospartum OR puerperium OR perinatal OR mother* OR maternal OR breastfeed* (Topic) and probiotic* OR prebiotic* OR synbiotic* OR psychobiotic* OR lactobacil* OR bifidobacter* OR saccharomyc* OR lactococc* OR bacillus OR pediococc* OR leuconostoc* OR "lactic-acid-bacteria" OR lacticaseibacillus OR lactiplantibacillus OR levilactobacillus OR ligilactobacillus OR limosilactobacillus OR oligosaccharid* OR fructo* OR galacto* OR oligosaccharide* OR xylooligosaccharide* OR Inulin OR fructans OR oligofructose OR "resistant starch" (Topic) and mental OR psychol* OR psychiatric* OR behavior* OR behaviour* OR wellness OR well-being OR wellbeing OR anxiety OR depression OR stress OR mood OR nervous* OR obsesi*(Topic) and trial* (Topic) |
| **Proquest Dissertations & Theses** | pregnan* OR prenatal OR gestation* OR pospartum OR puerperium OR perinatal OR mother* OR maternal OR breastfeed* (Topic) and probiotic* OR prebiotic* OR synbiotic* OR psychobiotic* OR lactobacil* OR bifidobacter* OR saccharomyc* OR lactococc* OR bacillus OR pediococc* OR leuconostoc* OR "lactic-acid-bacteria" OR lacticaseibacillus OR lactiplantibacillus OR levilactobacillus OR ligilactobacillus OR limosilactobacillus OR oligosaccharid* OR fructo* OR galacto* OR oligosaccharide* OR xylooligosaccharide* OR Inulin OR fructans OR oligofructose OR "resistant starch" (Topic) and mental OR psychol* OR psychiatric* OR behavior* OR behaviour* OR wellness OR well-being OR wellbeing OR anxiety OR depression OR stress OR mood OR nervous* OR obsesi* (Topic) and trial* (Topic) |

**Supplementary Material 3. Excludes studies and main reasons for exclusion.**

| **Author and Year** | **Title** | **Reason for Exclusion** |
| --- | --- | --- |
| Abdollahpour, 2023 | The effect of probiotic supplementation on episiotomy wound healing among primiparous women: a triple-blind randomized clinical trial | Authors don´t present information about our primary outcomes |
| Browne, 2021 | Probiotics as a treatment for prenatal maternal anxiety and depression: a double-blind randomized pilot trial | Pilot study and pregnant women with at least mild depressive symptoms and/or anxiety |
| El-Heis, 2023 | Maternal mood, anxiety and mental health functioning after combined myo-inositol, probiotics, micronutrient supplementation from preconception: Findings from the NiPPeR RCT | The intervention includes supplement enriched with myo-inositol, vitamin D, riboflavin, vitamin B6, vitamin B12 and zinc with probiotics together with standard folic acid, iodine, calcium, β-carotene and iron |
| Gupta, 2011 | A Double-Blind Randomized Clinical Trial for Evaluation of Galactogogue Activity of Asparagus racemosus Willd | The intervention is not a pro/pre/synbiotic or any dietary intervention microbiota-related |
| Movaghar, 2024 | The effects of synbiotic supplementation on blood pressure and other maternal outcomes in pregnant mothers with mild preeclampsia: a triple-blinded randomized controlled trial | Authors don´t present information about our primary outcomes |
| Slykerman, 2018 | Effect of early probiotic supplementation on childhood cognition, behaviour and mood a randomised, placebo-controlled trial | Primary outcomes are meassured in 11 y.o children |
| Dawe, 2020 | Probiotics and maternal mental health: A randomised controlled trial among pregnant women with obesity | In the sample there are women with depressive and/or anxious symptoms |
